# Calcium-Associated Proteins in Neuroregeneration

**DOI:** 10.3390/biom14020183

**Published:** 2024-02-02

**Authors:** Malwina Lisek, Julia Tomczak, Tomasz Boczek, Ludmila Zylinska

**Affiliations:** Department of Molecular Neurochemistry, Medical University of Lodz, 92-215 Lodz, Poland; malwina.lisek@umed.lodz.pl (M.L.); julia.tomczak.1@umed.lodz.pl (J.T.); tomasz.boczek@umed.lodz.pl (T.B.)

**Keywords:** calcium, neuroregeneration, CaM kinase II, GAP-43, oncomodulin, caldendrin, calneuron, NCS-1

## Abstract

The dysregulation of intracellular calcium levels is a critical factor in neurodegeneration, leading to the aberrant activation of calcium-dependent processes and, ultimately, cell death. Ca^2+^ signals vary in magnitude, duration, and the type of neuron affected. A moderate Ca^2+^ concentration can initiate certain cellular repair pathways and promote neuroregeneration. While the peripheral nervous system exhibits an intrinsic regenerative capability, the central nervous system has limited self-repair potential. There is evidence that significant variations exist in evoked calcium responses and axonal regeneration among neurons, and individual differences in regenerative capacity are apparent even within the same type of neurons. Furthermore, some studies have shown that neuronal activity could serve as a potent regulator of this process. The spatio-temporal patterns of calcium dynamics are intricately controlled by a variety of proteins, including channels, ion pumps, enzymes, and various calcium-binding proteins, each of which can exert either positive or negative effects on neural repair, depending on the cellular context. In this concise review, we focus on several calcium-associated proteins such as CaM kinase II, GAP-43, oncomodulin, caldendrin, calneuron, and NCS-1 in order to elaborate on their roles in the intrinsic mechanisms governing neuronal regeneration following traumatic damage processes.

## 1. Introduction

Traumatic brain injuries and neurodegenerative diseases can damage the central nervous system (CNS) and peripheral nervous system (PNS), leading to irreversible changes and causing functional deficits. Unlike in the CNS, damaged axons in peripheral nerves can be induced to regenerate in response to intrinsic cues and this process is triggered by a variety of inducers [1,2]. Proper axonal outgrowth in the adult CNS would be crucial for successful nerve regeneration and brain repair; however, an equilibrium between neuronal extrinsic and intrinsic factors is necessary to fully restore the injured neurons [3,4]. There are many identified mechanisms activated during neuronal injury, but due to complexity of the events regulating the subsequent regeneration, which involve multiple intrinsic signaling pathways, all the processes underlying the regeneration process have not been identified yet [5,6]. A wide variety of molecules are required in axonal regeneration, but because of the heterogeneity and functional diversity of neurons, they can also differentially affect the reactivation of specific tracts [7]. The efficiency of the regeneration process depends on the intrinsic properties of axonal outgrowth, the rate of the neuronal synthesis of proteins, cytoskeletal organization, axonal transport along the microtubules; all are critical for the regenerative response [3,8,9]. Of interest is the activation of numerous regeneration-associated genes (RAGs) after injury, and the participation of several transcription factors that are associated with regeneration [10,11,12].

Calcium ions, which physiologically play a very important function as second messengers, are also the inducers of cell death. It is well documented that the toxicity generated by increased, uncontrolled calcium levels in the cytosol after injury can lead to cell damage [13,14,15,16]. A significant component of the damage response process is a local translation in axons, which is essential for the regenerative effect. It enables the production of axonal regrowth molecules and induces regenerative pathways. For example, a moderate increase in Ca^2+^ is a factor that can stabilize the pool of F-actin required for the structural remodeling of spines. This multistep process involves the participation of several calcium-regulated proteins, including Ca^2+^/CaM kinase II and caldendrin [17,18,19]. Interestingly, calcium ions are also engaged in the restoration of neuronal homeostasis, which relies on calcium-regulated proteins expressed in the nervous system. Their specific role is determined by interactions with other functional proteins, which encompass Ca^2+^-regulated enzymes; proteins acting as Ca^2+^ sensors, represented by ubiquitous calmodulin (CaM) and CaM-like protein family; and Ca^2+^ buffers, which consist of many small, cytosolic proteins (i.e., parvalbumins, calbindins, calretinin) (for rev. [20,21]). Calmodulin appears to be the most recognized calcium sensor, and it can regulate over three hundred different targets with varying specific structural affinities [22,23,24,25] links Ca^2+^ signals to cellular functions through various effector proteins, including Ca^2+^/CaM-dependent kinases and phosphatases, or plasma membrane Ca^2+^-ATPase (PMCA) [26,27,28]. CaM binding by GAP-43 may also directly and indirectly affect regeneration process [29,30]. While many of CaM’s targets are located in neurons, we did not include CaM in this paper. Instead, we decided to focus on the less-known proteins that participate in axonal regeneration. In this short review, we focused on selected examples of proteins which may play a potential role as endogenous neuroprotectants. We provide an overview of the participation of Ca^2+^/CaM kinase II, GAP-43, oncomodulin (OCM), caldendrin, calneurons, and neuronal calcium sensor -1 (NCS-1) in neuroregeneration processes, discussing their possible impact on brain repair following pathological events.

## 2. CaMKII

Calcium/calmodulin-dependent protein kinase II (CaMKII) is a member of the serine/threonine kinases family, and involved in cellular calcium signaling throughout the body. CaMKII regulates a vast array of functions within the brain, heart, epithelia, and immune system [31,32,33,34]. In the brain, where was first discovered, CaMKII plays a crucial role in neurotransmission, synaptic plasticity, memory, and learning [35]. As a one of the amplest proteins in neurons, is expressed in four isoforms: α and β are identified mainly there, δ in the cardiovascular system, and γ ubiquitously, encoded by four diverse genes [31,35]. CaMKII is a holoenzyme consisting of 12–14 subunits, forming a hetero- or homomeric structure, determining its unique properties [31]. Each isoform’s structure is defined by four distinct elements: an N-terminal catalytic domain containing an ATP-binding site, a regulatory domain with a self-regulatory segment, multiple conserved sites of phosphorylation, a binding site for a Ca^2+^/CaM complex, a C-terminal domain consisting of a variable region, and an association motif required for subunits’ oligomerization [31,36].

Calcium influx initiates the binding of Ca^2+^ to CaM and as a complex bind to the CaMKII regulatory domain to induce a structural change, releasing the catalytic domain from the autoinhibitory region and exposing the substrate-binding site [36]. Ca^2+^/CaM binding results in autophosphorylation at T286 in CaMKIIα (Thr287 in CaMKIIβ, γ and ∂) in the autoinhibitory region, which enables phosphorylation of the neighboring activated subunit, causing Ca^2+^-independent CaMKII activity [31,36]. Interestingly, some CaMKIIγ subtypes, due to the nuclear localization sequence (NLS) signal, take part in shuttling the Ca^2+^/CaM complex to the nucleus to activate the cAMP-response element binding (CREB) protein in the brain [31].CaMKII, especially isoforms α and β, is a central coordinator of Ca^2+^ signal transduction in neurons and contributes to many physiological processes, like neurotransmitters metabolism, synaptic organization, long-term potentiation (LTP), and pathological processes, e.g., impaired learning, epilepsy, Alzheimer’s disease, ischemic stroke, and Parkinson’s disease [37]. The inability of axonal growth to occur in damaged neurons in the CNS is the main obstacle in the treatment of the diseases mentioned above [35]. It has been shown that calcium influx occurs immediately after axonal injury and promotes neuronal regeneration [38,39]. It is not surprising that CaMKII’s role in axonal regeneration has been proved in numerous studies.

The first reports demonstrated the promotion of neurite outgrowth in neuroblastoma lines when CaMKII was overexpressed or stimulated [40,41]. Likewise, pharmacological or genetic silencing inhibits CaMKII activity, leading to weakened axonal elongation in in vitro and in vivo studies or inducing the apoptosis and neuronal death of primary hippocampal neurons [35,42]. Moreover, activation of the kinase was relevant to the extension of prime neurons in vitro in embryonic CNS and mature PNS [35]. Axon regeneration, even of long-distance neurites, depends on the modulation of the cytoskeletal assembly at the nerve growth cone [43,44]. Since CaMKII colocalizes and operates with F-actin, a key cytoskeletal element, kinase activity may influence growth cone motility [45]. The inhibition of CaMKII destabilizes F-actin’s structure, and, in hippocampal neurons, overexpression leads to dendritic arborization, while silencing has the opposite effect [46,47]. Similarly, the repression of CaMKII activity leads to a reduction in F-actin length in the growth cone, and activation provokes the opposite effect [35]. In maturing neurons, CaMKIIβ manages filopodia motility and synaptogenesis, although this ability decreases with age or even acts in a contradictory way [47]. Moreover, CaMKIIβ’s presence is crucial not only in synapse formation, but also in spine maintenance for binding and bundling F-actin. During neuronal plasticity, the binding of Ca^2+^/CaM terminates CaMKIIβ/actin linkage, allowing unbundled F-actin to interact with other partners, causing the deep remodeling of filaments [36]. Consequently, inhibiting the dissociation of the CaMKIIβ/F-actin complex while simultaneously preserving kinase activity impairs structural and functional plasticity [18]. Moreover, CaMKII may contribute to axonal outgrowth by the phosphorylation of proteins associated with microtubules [48]. For instance, it can control the functions of cofilin, the protein modulating the cytoskeleton through actin depolymerization [49]. The other molecule Arc (activity-regulated cytoskeleton-associated protein), when co-expressed with CaMKII, caused higher axonal extension in neuroblastoma cells than in cells expressing CaMKII only [50]. This greater outgrowth was not achieved with Arc alone or with CaMKII inhibition, indicating that Arc only increases the enzyme effectiveness in neurite elongation. Among the cytoskeleton-associated proteins bonded by CaMKII and potentially mediating axonal growth are MAP-2 (Microtubule-Associated Protein 2), Tau, and Densin-180 [37].

Retinal ganglion cells (RGCs) are one of the most sensitive cells to changes in Ca^2+^ homeostasis during retinal ischemia or excitotoxicity. The first studies on CaMKII’s role in RGC survival and regeneration showed inconsistent results—chemically diminished CaMKII activity, under certain conditions, protects RGC, while in others it causes apoptosis and cellular hyperexcitability [51,52,53]. More recent research demonstrated that a loss of CaMKII activity in RGCs follows excitotoxicity and axonal damages [37]. CaMKII seems to be a crucial element in RGC maintenance, since kinase is highly phosphorylated in uninjured RGCs and the diminishing of CaMKII activity in vivo leads to RGC death. Under pathological conditions, such as NMDA-induced excitotoxicity and optic nerve crush (ONC), CaMKII activity declined. Interestingly, the enzyme reactivation caused the long-term protection of RGC somas and long-distance axon integrity within an optic nerve, preserving visual responses in the entire visual tract. Additionally, it was found that CaMKII/—CREB signaling plays a crucial role in RGCs’ survival. These results proved that kinase activity is critical for RGCs’ survival in pathological conditions and their maintenance in normal retina. Also, non-canonical Wnt/—CaMKII signaling appears to have an effect on adult mouse retina. The exogenous Wnt5 stimulation of RGCs promoted axonal regeneration after optic nerve injuries by inducing CaMKII/CREB signaling [54].

CaMKII has been implicated in several mechanisms that contribute to neuronal survival and recovery after ischemia. Previous reports have demonstrated evidence supporting the role of CaMKII activity in ischemic cell death, indicating that substantial neuroprotection can be achieved by inhibiting CaMKII. However, the broad inhibition of this pivotal kinase is challenging due to its involvement in various physiological processes like synaptic plasticity [55]. It was detected that the constitutive knockout of CaMKIIα provided protection against neuronal cell death following global cerebral ischemia in mice. Moreover, the long-term inhibition of CaMKII using tatCN21 or other inhibitors did not sensitize cortical cultures to ischemic insults [56]. Surprisingly, recent findings suggest that the autonomous activity of CaMKII, induced by T286 auto-phosphorylation, presents a promising target for post-insult neuroprotection after cerebral ischemia and potentially in other conditions, including glutamate excitotoxicity [57]. Additionally, CaMKIIδ and CaMKIIγ have been proposed as novel ischemia/reperfusion-induced genes that promote neuronal survival during ischemia. The upregulation of these CaMKII kinases activates the NF-κB signaling pathway, ensuring neuroprotection against ischemic injuries [58]. For a more in-depth understanding of the role of CaMKII in cerebral ischemia and its potential as a pharmacological target, readers are referred to [55,57].

## 3. GAP-43

Growth associated protein 43 (GAP-43) is an exclusively neuronal protein connected to nerve development and regeneration, synaptic plasticity, axonal pathfinding, and neurotransmission [30]. The expression of GAP-43 is highly elevated in neuronal growth cones during synaptogenesis, and after the completion of the process, it declines in most brain areas, except in regions involved in learning and memory, like the neocortex and hippocampus in the adult brain [59,60,61]. In neurons, GAP-43 prevails in axon terminals, enabling actin cytoskeleton modulation [62,63]. The importance of GAP-43 in neurodevelopment was verified by homozygous knockout mice, which died early in their neonatal period due to axonal misguiding and defects in cortical topography [59,62,63,64,65]. GAP-43 seems to be crucial not only for the axons of differentiating neurons but also for the centrosome, and a lack of GAP-43 expression led to the mislocalization of centrosome and mitotic spindles during brain growth, causing a significant reduction in the cerebellum [66].

**Figure 1:**
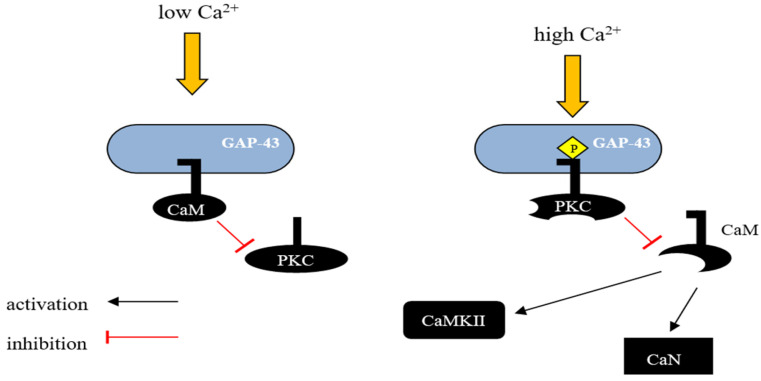
Schematic representation of GAP-43 activity regulation by calmodulin. GAP-43 can interact with CaM and the nature of this interaction depends on the intracellular concentration of Ca^2+^. In unstimulated neurons, where GAP-43 is present in large amounts, most of the CaM exists in a calcium-unbound form and is presumably located in the membrane fraction. In vivo, unphosphorylated GAP-43 binds to CaM at low Ca^2+^ concentrations in the cell, and this interaction inhibits the phosphorylation of Ser-41 by PKC, potentially protecting GAP-43 from activation in response to transient changes in the concentration of secondary messengers such as Ca^2+^, diacylglycerol, or arachidonic acid. However, when PKC is sufficiently activated, GAP-43 undergoes phosphorylation, leading to the release of CaM and blocking the possibility of its reassociation. This mechanism allows GAP-43 to remain in an active state even when the level of secondary messengers in the cell decreases. CaM—calmodulin, CaN—Ca^2+^ and calmodulin-dependent serine/threonine protein phosphatase 2B (Calcineurin), CaMKII—Ca^2+^/Calmodulin-dependent protein kinase II.

GAP-43 binds CaM more strongly at lower Ca^2+^ concentrations and releases it with a Ca^2+^ influx. Consequently, GAP-43 is considered as a temporary storage form place for CaM, which is was released at higher Ca^2+^ concentrations, sensitizing the downstream response (Figure 1) [29,67]. It was demonstrated that the phosphorylation of GAP-43 significantly alters its function in the cell. Phosphorylated GAP-43 stimulates actin polymerization and stability; however, it also has a role in presynaptic vesicle fusion in interacting with syntaxin, SNAP-25, and VAMP [68,69,70]. By influencing these molecular pathways, phosphorylated GAP-43 plays a role in facilitating various cellular processes, including axon guidance, axon outgrowth, neurotransmission, and synaptic plasticity [69,71,72,73]. GAP-43 can be phosphorylated by PKC, but also by other kinases on different residues, for instance, c-Jun N-terminal kinase (JNK) phosphorylates GAP-43 on Serine-96 and Serine-142 in growth cone membranes, facilitating axon elongation and regeneration [74].

GAP-43 plays a crucial role in guiding the axons of retinal ganglion cells as they navigate from the optic chiasm to the optic tract [75]. The optic chiasm is known as a critical decision point for retinal axons [76]. Although GAP-43 is not essential for axonal outgrowth, it is crucial for the proper pathfinding of growth cones [76]. During the formation of the optic tract, GAP-43 facilitates the interaction of retinal axons with the lateral diencephalon [75]. In vivo studies with GAP-43 knockout mice revealed a failure to develop the anterior commissure, hippocampal commissure, and corpus callosum [64]. GAP-43 is also vital for the normal development of serotonergic innervation in the forebrain, playing a necessary role in the axonal outgrowth and terminal arborization of serotonergic axons originating from the raphe nuclei [77]. In Gap-43-null mice, serotonergic innervation in the cortex and hippocampus was almost abolished, as is implicated in conditions such as schizophrenia, depression, anxiety, and autism [77]. Interestingly, the inhibition of nitric oxide synthase (NOS) has been demonstrated to induce GAP-43 expression in the developing retina of postnatal rats [78]. It should be mentioned that, contrary to its regenerating effect after optic nerve injury, the overexpression of GAP-43 was found to be deleterious for RGCs in an experimental chronic laser-induced chronic intraocular pressure (IOP) elevation injury mimicking glaucoma [79].

GAP-43 plays an important role in nerve regeneration following injury and GAP-43 mRNA overexpression is closely associated with an improved regenerative capacity [80]. Elevated GAP-43 expression is also linked to axonal sprouting in the barrel cortex after a stroke [81]. Additionally, higher GAP-43 levels are connected to the optogenetic-induced functional recovery of the primary motor cortex after stroke [82]. In rodent stroke models, the use of antisense oligonucleotides targeting GAP-43 was found to counteract the enhancement of functional recovery induced by basic fibroblast growth factor [83]. These findings underlined the crucial role of GAP-43 in neuronal recovery post-injury. Knockdown studies further emphasize the indispensable role of GAP-43 in nerve regeneration [84,85]. In adult rodents post injury, climbing fibers preserved high GAP-43 abundance and structural plasticity [86]. An increased GAP-43 level caused F-actin accumulation and subsequent neurite sprouting in primary sensory neurons and in Purkinje neurons [87,88]. It was proved, in studies involving rodent models of stroke and traumatic brain injury (TBI), that the activation of nerve growth factor (NGF) and brain-derived neurotrophic factor (BDNF) signaling promotes neuroprotection, synaptogenesis, and neurogenesis [89,90,91]. Optogenetic stimulations post-stroke resulted in a beneficial increase in NGF, BDNF, and GAP-43 levels [82]. Additionally, GAP-43 was identified as an essential mediator of BDNF-driven neuroprotection [92].

## 4. Calcium-Binding Proteins

**Figure 2:**
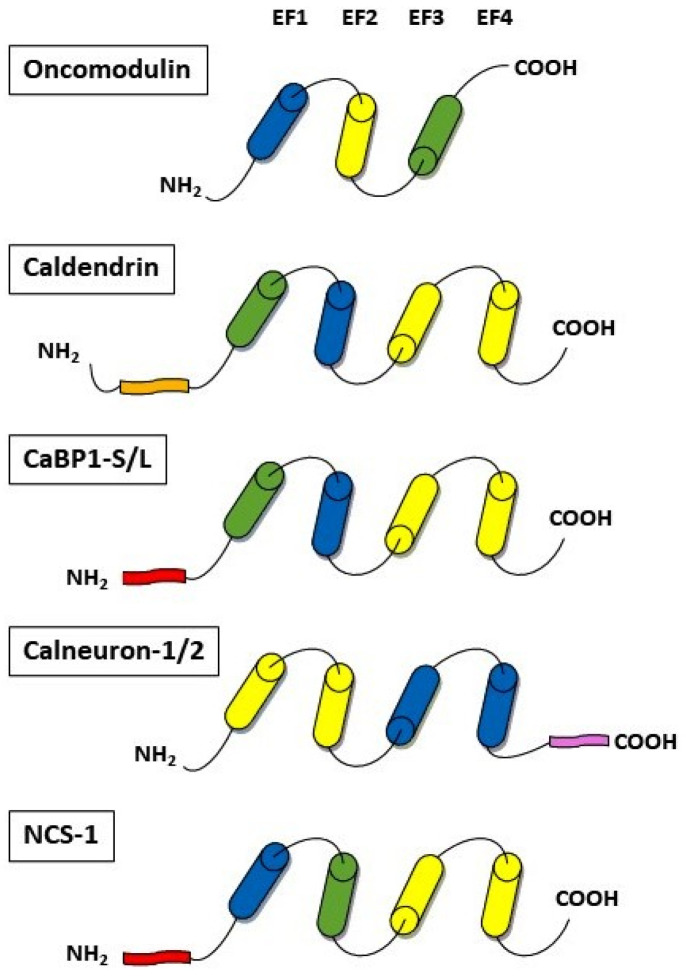
Schematic diagram showing the domain structure of calcium-binding proteins. Oncomodulin possesses three EF-hand domains. EF1 cannot bind calcium ions (*in blue*), while EF2 can bind a single Ca^2+^ (*in yellow*) and is characterized by a higher specificity for calcium ions, and EF3 accommodates both Ca^2+^ and Mg^2+^ (*in green*). Caldendrin possesses only two functional (EF3 and EF4, *in yellow*) and two atypical (EF1 and EF2) domains. EF1 can bind bothMg^2+^ and Ca^2+^ (*in green*), whereas EF2 is non-functional (*in blue*). The N terminus contains a unique proline-rich domain with multiple PxxP motifs (*in orange*). The two shorter isoforms of caldendrin, CaBP1-S and CaBP1-L, differ in their sequence and are N-terminally myristoylated, which determines their localization in the membranes. Calneurons have active EF1 and EF2 (*in yellow*) and inactive EF-hands 3 and 4 (*in blue*). A 38 amino acid tail at the C-terminus (*in pink*) localizes these proteins to membranes, particularly of the trans-Golgi network. In NCS-1, the first EF-hand cannot bind Ca^2+^ (*in blue*), EF2 is a Mg^2+^- and Ca^2+^-binding domain (*in green*), and EF3 and EF4 can bind Ca^2+^ (*in yellow*). The N-terminal myristoylation site (*in pink*) promotes NCS-1 binding to the cell membranes.

Ca^2+^ serves as a critical second messenger in neuronal signaling and can exert both positive and negative effects on neurite regeneration, involving a variety of proteins that transduce Ca^2+^ signals for neurite growth. Several identified Ca^2+^-binding proteins play a crucial role in controlling Ca^2+^ concentrations and may be essential for locally manipulating cellular calcium to facilitate neuronal repair. However, their neuron-specific expression and processes triggered following traumatic damage can lead to variations in their neuroregenerative properties and neurite growth dynamics. Within the category of calcium-binding proteins, there is a subset of EF-hand proteins related to calmodulin (CaM), which are highly expressed in the neuronal cell types of the brain. The EF-hand (helix-loop-helix motif) is one of the most frequently observed domains, and over 1000 have been identified based on their unique sequence signatures [93]. Interestingly, EF-hand proteins are composed of one or more of these domains. In Figure 2, we have depicted the schematic structure of selected examples of the proteins that may play a potential role in the regulation of neuronal repair.

### 4.1. Oncomodulin

Oncomodulin (OCM) is a small Ca^2+^-binding protein (CaBP) belonging to the parvalbumins family, which are classical, small, mostly cytosolic and EF-hand-containing motifs molecules [94]. The EF-hand motif is characterized by an α-helix-loop-α-helix arrangement spanning approximately 30 amino acids, responsible for Ca^2+^ binding and triggering a conformational change leading to the activation or inactivation of target proteins [95]. OCM, as a member of the parvalbumin family, displays distinct characteristics in its ability to bind metal ions and sense calcium, resulting in functional differences when compared to other family members. Of the three domains in oncomodulin, EF1 cannot bind calcium ions due to the absence of key residues, but plays a vital role in OCM’s structural stability, EF2can bind a single Ca^2+^ and is characterized by a higher specificity for calcium ions, while EF3 accommodates both Ca^2+^and Mg^2+^ (Figure 2). Upon exposure to calcium ions, OCM undergoes distinctive structural changes not observed in other parvalbumins. OCM features a cation binding site that accommodates both calcium and magnesium, with one being more Ca^2+^-specific. It is proposed that OCM might function as a calcium sensor or modulator under specific physiological conditions, rather than solely acting as a Ca^2+^ buffer [95].

OCM’s function appears ambiguous and cell-type-dependent when distinguishing it from other EF-hand CaBPs. Mammalian OCM expression is unique and confined to specific inner ear hair cells and specific immune cells [96,97,98]. In adult mammals, OCM can act as a Ca^2+^ buffer and likely influence outer hair cell (OHC) motility mechanisms, contributing to sensory hair cell function and playing a crucial role in maintaining hearing function and health [99,100]. Additionally, OCM impacts the development of OHCs and their neonatal afferent innervation by modulating Ca^2+^ activity and regulating the expression of key channels and receptors [101,102]. Moreover, OCM has been thought to serve as a factor secreted by macrophages and neutrophils to facilitate nerve regeneration [97].

In this review we want to focus on the most important function of OCM, which is linked to its potential to regenerate injured axons. Generally, the central nervous systemand peripheral nervous system exhibit distinct abilities in terms of nerve regeneration. While, in the PNS, axon regeneration can occur spontaneously, in the CNS, after injury, such a process is inhibited by many extrinsic and intrinsic factors [103,104,105]. Moreover, itsintrinsic regenerative ability is self-limiting, depending on the characteristics and type of injury. Axonal regeneration can be enhanced by a preconditioning injury and the response of the innate immune system [106,107,108]. A preconditioning injury can create a remarkable ability of the CNS to regenerate axons even with the presence of inhibitory factors within its environment, including transcriptional control mechanisms that regulate intrinsic axon growth ability [109,110]. This effect is initiated by a previous injury to peripheral branches outside the CNS, as evidenced by studies illustrating the regeneration of axons in the CNS after a previous injury in the dorsal root ganglia (DRG) of peripheral branches. DRG preconditioning injuries can induce chemokine signaling, recruiting and activating perineuronal monocytes and macrophages [111,112]. The induction of an inflammatory reaction within the DRG can enhance the ability of sensory neurons to regenerate their central axon branches through their dorsal roots [113]. While the exact interaction between accumulating immune cells and neurons that promotes regeneration remains unclear, the OCM expressed by macrophages and neutrophils emerges as a potential key player [114]. The research provides compelling evidence supporting OCM’s role in amplifying the intrinsic capacity of DRGs for axonal regeneration by influencing neuronal gene expression [109,115,116]. The studies also raise the possibility that OCM may exhibit chemotactic activity or modulate the expression of monocyte chemokines (e.g., CCL2) and their signaling within the DRG [111,112,117].

Interestingly, introducing OCM through a specialized delivery system complex via intra ganglionic injection leads to a notable long-range regeneration of dorsal column sensory axons, extending beyond spinal cord lesions [118]. Furthermore, based on studies on the regeneration of retinal ganglion cell axons, OCM has also been identified as a macrophage-derived growth factor that promoted nerve regeneration following induced ocular inflammation [116,119]. Significantly, it has been established that, in addition to macrophages, neutrophils play a crucial role as a major source of OCM, actively promoting axon regeneration and serving as primary responders during inflammation and after injury [120,121]. The findings underscore the predominant role of OCM in mediating this regenerative effect, even in the presence of various proinflammatory agents that positively impact optic nerve regeneration [98].

As was mentioned, oncomodulin effects are highly specific to certain neurons, particularly DRG sensory neurons and retinal ganglion cells (RGCs) [116,119,122]. Research indicates that OCM does not induce neurite outgrowth activity in cultured cortical neurons or spinal cord-derived neural stem cells (NSCs). The mechanisms underlying its selective neuroregenerative activity are likely associated with the tissue-specific expression of the high-affinity receptor ArmC10 (armadillo-repeat protein C10). OCM functions through ArmC10 to enhance the regeneration of the optic nerve and peripheral nerves, facilitating the regeneration of spinal cord axons. ArmC10 is prominently expressed in human-induced pluripotent stem cell-derived sensory neurons, and exposure to OCM has been demonstrated to modify gene expression and promote neurite outgrowth in these cells. Additionally, ArmC10 is expressed in human monocytes, and OCM has been found to increase the expression of immune modulatory genes within these cells [123].

The detailed mechanisms by which OCM exerts its effects remain unknown. In RGCs OCM operates within cellular signaling pathways, notably the CaMKII pathway [119]. The initiation of nerve regeneration involves a rapid influx of sodium and calcium ions, triggering essential cellular processes. CaMKII emerges as a central coordinator in Ca^2+^ signal transduction, activating various protein kinase pathways and transcription factors, including CREB [105,124].

Simultaneously, the orchestration of the second messenger signaling pathways comes into play, instigating the generation of cyclic adenosine monophosphate (cAMP), a pivotal factor critical to the intricate process of regeneration. Elevated cAMP levels prompt the translocation of the oncomodulin receptor in retinal ganglion cells, RGCs, to the cell surface, facilitating the binding of secreted OCM [98,123]. The functionality of OCM in RGCs is intricately linked to the phosphorylation and activation of CREB. CREB, in turn, governs a considerable number of neuronally enriched coding genes associated with a spectrum of intracellular processes, including proliferation, differentiation, survival, long-term synaptic potentiation, neurogenesis, and neuronal plasticity [125,126,127,128]. Moreover, CREB’s association with the selective transcription of “regeneration-associated genes” (RAG) induced by the conditioning lesion reveals its significance in the regulatory network orchestrating gene expression [129]. Importantly, studies demonstrate that the expression of a constitutively active CREB protein can actively promote the regeneration of dorsal column sensory axons in vivo, underscoring CREB’s functional importance in the context of regeneration [129,130]. In DRGs, following preconditioning sciatic nerve injury, oncomodulin actively contributes to the upregulation of neuropeptide-related genes belonging to selected RAG sets, including Npy, Gal, Vip, Sprr1a, Gap-43, Ankrd1, Csrp3, and Reg3b, all of which exhibit neurite outgrowth activity [118]. A slightly less pronounced increase was detected in the expression of genes associated with the extracellular matrix and cell adhesion (Adam8 and Itga7), and cytokine/growth factors (Il6, En1, Gfra1), in response to OCM [118]. This suggests a multifaceted influence of OCM on the cellular microenvironment, extending its impact beyond direct neurite outgrowth regulation. Furthermore, OCM demonstrates synergistic potential with other factors, such as stromal cell-derived factor 1 (SDF1), amplifying its regenerative effects. This small chemokine is another inflammatory factor reported to promote optic nerve regeneration that is also highly expressed in macrophages. The combination of OCM, SDF1, and a cAMP analog not only replicates but also surpasses the effects of intraocular inflammation on optic nerve regeneration, presenting a promising avenue for enhancing regenerative outcomes [131]. OCM’s impact on peripheral sensory neurons, similar to the optic nerve, is intricately linked to SDF1. Their combined application efficiently expedites the regeneration of both peripheral nerve and spinal cord axons [105].

### 4.2. Caldendrin

Among the interesting Ca^2+^-binding proteins is CaBP1, which, due to alternative splicing, forms three variants: caldendrin, CaBP1-S, and CaBP1-L [132,133]. Caldendrin has been shown to be highly expressed in different neuronal cell types, including the brain, retina, and inner ear [134,135]. Specifically, it is present in the synapses of cerebral cortical neurons, the cerebellum, hippocampus, and thalamus, as well as in the postsynaptic density of spine synapses [136,137]. Caldendrin has a unique bipartite structure with high homology to calmodulin, a ubiquitous EF-hand calcium sensor protein (Figure 2). However, in contrast to calmodulin, other Ca^2+^ sensor proteins have more specialized functions. Caldendrin possesses only two functional domains (EF3 and EF4) and two atypical domains (EF1 and EF2) [138]. The EF1 hand can bind Mg^2+^ with high affinity, whereas EF2 is non-functional. Two shorter isoforms of caldendrin detected in rats and humans differ in their sequences and are N-terminally myristoylated [133,137,139]. These proteins are mainly localized in the cytosol but are also present in the Golgi structures [140]. It should be underlined that caldendrin exhibits a high degree of conservation in humans, rats, and mice [141].

Caldendrin has been suggested to be involved in the processing of synaptic Ca^2+^ signaling [142]. A long-lasting increase in intracellular Ca^2+^ levels, either due to an influx through Ca^2+^ channels or a release from Ca^2+^ stores, is neurotoxic. However, some channels can be rapidly inactivated by the Ca^2+^-dependent inactivation process (CDI), which is a negative feedback mechanism important for regulating Ca^2+^ entry under both physiological and pathological conditions [143,144,145]. Physiologically, caldendrin increases the Ca^2+^influx throughL-type voltage-gated Ca^2+^ channels—Ca_v_1.2 and Ca_v_1.3—by interacting with their C-terminal domain [146,147,148,149,150]. In contrast, CaBP1 enhances the inactivation of Ca_v_2.1 channels (P/Q-type) by interacting with the CaM-binding domain of the channel [151]. Additionally, the transient receptor potential channel, TRPC5, implicated in neurite extension and the growth cone morphology of hippocampal neurons, is negatively controlled by CaBP1 [152]. An increase in cytosolic Ca^2+^ levels to the micromolar range subsequently initiates many neuronal processes, including the release of neurotransmitters, gene transcription, and the growth of neurites. This plays an important role in synaptic plasticity and memory formation [149]. It has been described that caldendrin may compete with calmodulin for binding to the same regulatory sites in many CaM-regulated proteins [150]. Of particular interest is the observation that caldendrin, along with its shorter CaBP1 forms, can inhibit IP3 binding to inositol 1,4,5-trisphosphate receptors (IP3Rs) in a Ca^2+^-independent manner, thus blocking Ca^2+^ release from the endoplasmic reticulum [153,154,155]. This effect may differentially regulate the function of various effectors and shape neuronal Ca^2+^ signals.

Caldendrin can also support the cytoskeleton network by stabilizing F-actin within the network’s spines through its interaction with cortactin, a protein implicated in actin filament nucleation and branching. This regulation contributes to spine stability and participates in the development of long-term memory and its storage [142]. Interestingly, cortactin activation appears to be caldendrin-specific and CaM-independent. The dynamics of spine morphology are attributed to the actin cytoskeleton that is highly concentrated in spines. However, the transport of organelles and their anchoring at dendritic spines also depend on F-actin-linked myosins [156]. Recently, it was demonstrated that in hippocampal neurons, caldendrin can associate with several myosin family members, including myoVa, myoVb, and myoVI, in a Ca^2+^-dependent manner [157]. Because myoV transports smooth endoplasmic reticulum into activated spines, Ca^2+^/caldendrin may replace Ca^2+^/CaM in myosins, thereby inhibiting further motility. This is of great importance, as the proper synaptic targeting of smooth endoplasmic reticulum is crucial for the formation of the spine apparatus in dendrites during neuronal processes such as long-term potentiation (LTP).

A number of studies using caldendrin knockout animals have shown a more intensive regenerative growth of neurites, confirming the inhibitory action of this protein in the regeneration and elongation of neurites [158,159]. It is suggested that caldendrin may influence an initial phase of regeneration by altering the transcription of genes that promote axon outgrowth [159,160].

A crucial role in coordinating neuronal processes involves the formation of protein complexes named signalosomes, which are formed by scaffold proteins at specific subcellular locations. A notable example is a family of A-kinase anchoring proteins (AKAPs) that bind to protein kinase A (PKA) and other secondary messenger-regulated enzymes [161]. One of the essential elements for signal transduction is the protein AKAP79/150, which links receptors, channels, and other signaling proteins to physiological substrates and has been associated with the regulation of neurite growth [162]. At postsynaptic sites in neurons, AKAP79/150 can form complexes with protein kinases A and C, calcineurin, calmodulin, phosphatidylinositol 4,5-bisphosphate, and also with caldendrin [163]. It was shown that calmodulin and caldendrin compete for a binding site on AKAP79/150, but due to their different binding characteristics, these proteins might play complementary roles [164]. Caldendrin preferentially binds to AKAP 79/150 and forms a stable complex under resting conditions, while competing with CaM at high Ca^2+^ concentrations. Since caldendrin, CaM, and AKAP 79/150 can also interact with Ca_v_1.2, the binding of CaM promotes CDI, but the binding of caldendrin stabilizes the open state of the calcium channel.

One of the signaling pathways in neurons involves interactions between initial activity-dependent molecular changes at the synapse and the subsequent regulation of gene transcription in the nucleus. An interesting example of such communication is the contribution of Jacob, a protein messenger abundantly expressed in the brain, whichconnects NMDA-receptor-derived signalosomes to the transcription factor CREB [165,166,167]. The activation of synaptic NMDARs induces the expression of pro-survival genes, but the activation of extrasynaptic NMDARs initiates the expression of cell death genes [168]. The overexpression of Jacob triggers the expression of genes that induce neurodegeneration, whereas the nuclear knockdown of Jacob increases the phosphorylation of CREB and protected neurons from an extrasynaptic NMDA receptor-induced loss of synaptic contacts and neuronal cell death [169]. Caldendrin was shown to bind to Jacob’s nuclear localization signal in a Ca^2+^-dependent manner, and this interaction enabled the proper formation of the signalosome, representing a powerful regulatory mechanism of synapse-to-nucleus communication. Ultimately, significant changes in the morphology of the dendritic tree were generated [169]. 

In light of the above reports, caldendrin appears to be a somewhat enigmatic protein that can not only regulate neuronal cell fate on several levels of Ca^2+^-dependent neuronal signaling but also can play a modulatory role in degeneration/regeneration events. Little is known about the function and localization of both shorter CaBP1 splice variants. Structurally, they differ from caldendrin since they possess an N-myristoylation motif, by which they could be anchored to vesicular membranes. Moreover, the very low abundance of the shorter CaBP1 forms in the brain could result in different cellular localization, as well as diverse functions in the transduction of Ca^2+^ signals. It was suggested that, in contrast to CaBP1-S and -L, caldendrin may fulfill specific requirements for somato-dendritic Ca^2+^ signaling in limbic and cortical neurons, while its short variants are more closely related to classical NCS proteins [170]. Another study has shown that CaBP1-S is localized at or near the plasma membrane, whereas CaBP1-L is most likely associated with cytoskeletal structures [135]. Thus, the splice forms may have distinct localization patterns, producing further diversification among CaBPs, and could be an important mechanism for targeting the effector proteins of different cellular compartments, as well as inducing diverse responses to Ca^2+^ signals.

### 4.3. Calneurons

Within the CaBP family, there also exists an interesting calciumsensor subfamily of proteins consisting of calneuron 1 (CaBP8) and calneuron 2 (CaBP7), which are abundant in the brain and exhibit developmental changes in expression [135,141,171]. They have 64% similarity and are present at high levels in distinct regions of the adult mammalian brain [172,173]. 

It is noteworthy that calneurons are highly conserved among different species, exhibiting 100% identity at the amino acid level between mice, rats, monkeys, and humans. This underscores their crucial cellular function. Both calneurons are distributed across various subcellular fractions, primarily localized in Golgi membranes, cytoplasm, and vesicles. In the mouse brain, calneuron 1 was found in significant amounts in the cerebellum, hippocampus, and cortex [174]. This suggests its potential role in signal transduction, which could be crucial for memory and learning processes. Given the restricted expression of calneuron-2, this implies rather it has specific functions within a subset of neurons.

Additionally, calneurons exhibit sequence homology with calmodulin. In contrast to other calcium-binding proteins (CaBPs), calneurons display a distinct pattern of EF-hand inactivation, featuring active EF-hands 1 and 2 and inactive EF-hands 3 and 4 [171] (Figure 2). Calneurons possess a unique 38 amino acid extension in their C-terminal domains, which imparts a strong membrane association, particularly with the trans-Golgi network (TGN) [175]. It also enables calneurons’ interaction with unique targets and can be used to target proteins to the TGN [176,177]. Both proteins demonstrate the highest Ca^2+^-affinity among all EF-hand Ca^2+^sensors, with dissociation constants of 230 nM for calneuron-1 and 180 nM for calneuron-2 [173]. 

Less is known about functions of calneuron-1 and calneuron-2 [138]. As of publication, they have been found to regulate the activity of phosphatidylinositol 4-OH kinase IIIβ (PI4KIIIβ), which catalyzes the local synthesis of the phosphoinositides necessary for vesicle assembly in the trans-Golgi network (TGN) [178]. The activity of PI4KIIIβ at the Golgi membrane is a crucial initial step in trans-Golgi network-to-plasma-membrane trafficking. A primary and central regulator of PI4KIIIβ activity is NCS-1, a neuronal calcium sensor protein responsible for the rapid transduction of Ca^2+^ signals. NCS-1 is involved in numerous physiological neuronal functions, including exocytosis, the regulation of calcium channels, nuclear Ca^2+^ regulation, neurite outgrowth, and neuroprotection, as well as axonal regeneration in response to neuronal damage [155,179,180]. Therefore, it was interesting to find that both calneurons can directly associate with PI4KIIIβ both in vitro and in vivo in a Ca^2+^-independent manner [173]. Moreover, this interaction has the opposite effect compared to NCS-1, as calneurons inhibit the enzyme. Consequently, this leads to a reduction in the lysosomal pool of PI_4_P, a significant precursor of phosphatidylinositol 4,5-bisphosphate (PIP_2_), a prominent inositol lipid signaling molecule. Serving as a regulator of PI4KIIIβ, calneuron-2 plays a role in the biogenesis of vesicular carriers originating from the trans-Golgi network (TGN) in neuronal cells [173]. 

Recently, it was reported that the association of calneuron-2 with lysosomes during cytokinesis regulates their clustering. By modulating phosphatidylinositol 4-phosphate levels, it can control cytokinesis in mammalian cells [178]. Calneuron-2 has been identified as a factor necessary for the normal completion of cytokinesis in mammalian cells. Its suppression results in cytokinesis failure after the normal segregation of chromosomes [181].

Interesting data have shown that, in striatal cells, calneuron-1 can modulate the G-protein coupled receptors (GPCR) heteromers: adenosine A_2A_ receptor (A_2A_R)-dopamine D_2_ receptor (D_2_R) [182]. These heteromers seem to be a crucial target for the treatment of Parkinson’s disease and other neuropsychiatric disorders. It has been demonstrated that varying intracellular Ca^2+^ levels exert a differential modulation of A2AR-D2R heteromer-mediated adenylyl cyclase activity and MAPK signaling. The selective interaction of calneuron-1 with the A2AR-D2R heteromer under high Ca^2+^ levels resulted in a very low activation of both MAPK signaling and cAMP production.

Another potential relationship between calneuron-1 and the regulation of GPCRs was demonstrated with the cannabinoid CB1 receptor. This receptor, highly expressed in CNS neurons, is coupled to Gi proteins [183]. The interaction of the GPCR with calneuron-1 was found to be calcium-dependent, and the binding of CB1R to calneuron-1 completely blocked Gi-mediated signaling. The aforementioned studies indicate that calneurons can provide subtle regulatory control of Ca^2+^ signals in various physiological processes, playing an essential role in the integration of cellular mechanisms, including neuronal transmission, Golgi-to-plasma-membrane trafficking, and normal cytokinesis. While less is known about the potential involvement of calneurons in neuronal disorders, recently, similar to caldendrin, they have been identified as a risk gene for schizophrenia [184,185,186].

### 4.4. Neuronal Calcium Sensor-1 

Neuronal calcium sensor-1 (NCS-1) is a member of the NCS superfamily, characterized by four EF-hand domains (Figure 2). As with other members, the first EF-hand is unable to bind Ca^2+^, EF-2 serves as a Mg^2+^/Ca^2+^-binding domain, and the C-terminal domain contains Ca^2+^-binding EF-3 and EF-4 [187]. NCS-1 features an N-terminal myristoylation site facilitating its binding to cell membranes [188]. Ca^2+^ binding to NCS1 causes a structural rearrangement, modulating its affinity for target molecules [189]. In the brain, NCS-1 is widely expressed, with the highest abundance observed in neuronal tissues, particularly in the cortex, as well as in the hippocampus and dorsal root ganglion cells [190,191]. The specific expression patterns of NCS-1 in various brain regions, cell types, and subcellular areas can influence the availability of its unique target proteins. Consequently, its diverse responses to changes in neuronal Ca^2+^ may impact downstream signaling pathways in the brain [192,193,194,195]. Due to its high Ca^2+^-binding affinity (K_d_ for Ca^2+^ 200 nM), NCS-1 can respond to Ca^2+^ fluctuations above resting levels. This characteristic suggests its potential involvement in protecting neurons from damage under pathological conditions [196,197].

To date, NCS-1 has been demonstrated to regulate various cellular functions, encompassing exocytosis, neurite outgrowth, neuroprotection, and axonal regeneration [195,198]. The functional diversity of NCS-1 arises from its interaction with numerous downstream targets. These include the binding to and regulation of the Ca_V_2.1 of VGCCs, the enhancement of inositol 1,4,5-trisphosphate receptor activity, and the spatial and temporal control of phosphatidylinositol 4-phosphate levels through the activation of phosphatidylinositol 4-kinase III-β (PI-4Kβ). Additionally, NCS-1 contributes to the desensitization of dopamine type-2 receptors, among other pathways [199,200,201,202,203,204,205,206,207]. An intriguing relationship has been proposed regarding a molecular switch in the Ca^2+^ regulation of PI-4Kβ activity by calneurons and NCS-1 in Golgi membranes. Calneurons at low Ca^2+^ levels suppress PI-4Kβ activity and dominate in the regulation of this enzyme. As Ca^2+^ increases, the formation of the NCS-1/PI-4Kβ complex is promoted, potentially allowing for the override of the inhibition of PI-4Kβ activity imposed by calneurons [173]. Moreover, a transient receptor potential channel with Ca^2+^/Na^+^ permeability, TRPC5, known for its inhibitory role in neuronal outgrowth, can form a protein complex with NCS-1 in rat brains [208]. It has been suggested that NCS-1 acts as a Ca^2+^ sensor, enabling TRPC5 to link Ca^2+^ transients to their effects on growth cone extension. Neurite extension and branching are affected by the activity-dependent modulation of intracellular Ca^2+^,and a direct interaction between NCS-1 and TRPC5 had a positive impact on TRPC5 and Ca^2+^ influx. Given that NCS-1 differentially modulates Ca^2+^ currents in the soma and growth cones of regenerating neurons, it may serve as a crucial regulator to facilitate growth cone development [209]. While NCS-1 does not function as a classical transcription factor, it can regulate nuclear Ca^2+^ signaling, thereby indirectly influencing gene expression. The activation of cytosolic CaMKII-α, a major regulator of transcription factors such as CREB, is facilitated by NCS-1. This activation of Ca^2+^-dependent CaMKII-α signaling may directly and/or indirectly lead to an increase in brain-derived neurotrophic factor (BDNF) levels [210,211].

Several studies have suggested that NCS-1 serves as a Ca^2+^-dependent survival-promoting factor, as its expression increases in injured neurons, providing protection against cell death. It has been demonstrated that NCS-1 can enhance the resistance of neurons to stressors, i.e., oxidative stress or trophic supplement withdrawal, thereby exerting a protective effect [180,212]. Furthermore, NCS-1 is up-regulated in response to axonal injury in adult motor neurons, preventing apoptosis. An additional neuroprotective role for NCS-1 has been proposed, demonstrating its ability to promote the long-term survival of cultured neurons through PI3-K–Akt signaling pathways. This neuroprotection is partly mediated by glial cell line-derived neurotrophic factor (GDNF), and NCS-1 is up-regulated in response to axonal injury [212].

The overexpression of NCS-1 induces neurite sprouting and is associated with increased phospho-Akt levels. The inhibition of the PI3K/Akt signaling pathway abolishes neurite sprouting [180]. NCS-1 is involved in the regulation of cell survival pathways, not only through Akt and PI3K, but also through PI-4Kβ, whose activity is regulated by changes in intracellular Ca^2+^ concentration. Using cortical neurons as a model, it has been demonstrated that NCS-1 knockdown leads to changes in gene expression, predominantly resulting in the hyperexcitation of neurons, especially GABAergic neurons [213]. Moreover, under ischemia-like conditions and reoxygenation, neurons with reduced NCS-1 expression exhibit increased sensitivity to damage, characterized by an elevated expression of pro-apoptotic genes and a down-regulation of protective genes.

Numerous experimental studies have demonstrated that the processes occurring in growth cones and soma in developing and regenerating neurons can employ similar molecular mechanisms. The proper extension and branching of neurites require an optimal window of Ca^2+^ levels, and these processes are dynamically influenced by the activity and/or presence of a set of Ca^2+^-binding proteins essential for axonal outgrowth. While the significance of NCS-1 as a facilitator enabling the rapid transduction of Ca^2+^ signals is well recognized, the precise cellular and molecular mechanisms that regenerate neurons are still to be determined.

## 5. Conclusions

**Figure 3:**
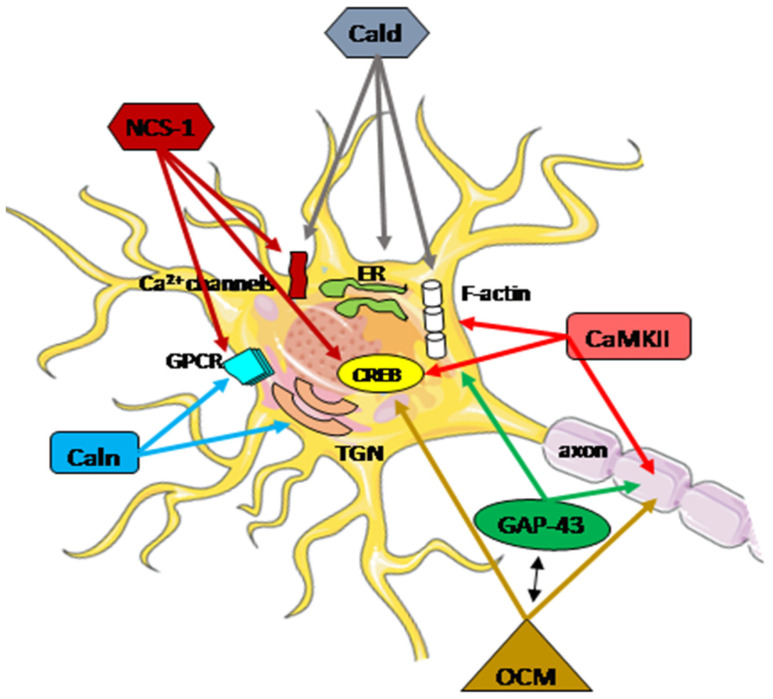
Schematic presentation of the regulatory function of Ca^2+^-associated proteins in neuron repair. A subtle regulatory control of Ca^2+^ signals plays a crucial role in integrating cellular mechanisms, and the cooperation of all potentially interacting partners is essential for specific neuronal functions. These functions encompass neurotransmitter release, proper neuronal transmission, activity-dependent gene transcription, endoplasmic reticulum (ER) targeting, Golgi-to-plasma-membrane trafficking, axonal growth, and cytokinesis. Arrows within the figure indicate potential direct sites and/or processes where specific Ca^2+^-associated proteins may be engaged. The activation or inhibition of neuron repair depends on the presence of partner proteins and the Ca^2+^ level. CaMKII actively participates in axon regeneration, colocalizes, and interacts with F-actin, activating the cAMP-response element-binding (CREB) protein. Phosphorylated GAP-43 stimulates F-actin polymerization and stability, leading to F-actin accumulation. GAP-43 also plays a pivotal role in axon outgrowth, neurotransmission, and synaptic plasticity. Oncomodulin (OCM) facilitates axon regeneration by operating within cellular signaling pathways, notably the CaMKII pathway, and is linked to the phosphorylation and activation of CREB. OCM contributes to the upregulation of selected RAGs, including Gap-43. Caldendrin (Cald) and two shorter CaBP1 forms regulate the Ca^2+^ influx through Ca^2+^ channels, including L-type and P/Q-type VGCC, and also TRPC5, which is implicated in neurite extension and growth cone morphology. Furthermore, Caldendrin, in a Ca^2+^-independent manner, could inhibit IP_3_ binding to inositol 1,4,5 trisphosphate receptors (IP3Rs), thereby blocking Ca^2+^ release from the ER. It also supports the cytoskeleton network by stabilizing F-actin within the spines.Calneuron-1 and Calneuron-2 (Caln) regulate the activity of phosphatidylinositol 4-OH kinase IIIβ, catalyzing the local synthesis of phosphoinositides necessary for vesicle assembly in the trans-Golgi network (TGN). Calneuron-1 can modulate G-protein-coupled receptor (GPCR) heteromers, such as adenosine A2A receptor (A2AR)-dopamine D2 receptor (D2R) and, as recently shown, cannabinoid CB1 receptor. Neuronal calcium sensor-1 (NCS-1) binds to and regulates the Ca_V_2.1 of VGCCs and TRPC5, enhancing the activity of IP3Rs and PI-4Kβ in Golgi membranes. Additionally, it contributes to the desensitization of D2Rs. The NCS-1-induced activation of cytosolic CaMKII-α can regulate CREB.

The highly diverse nature of the Ca^2+^ signaling in the nerve system is well established. At every stage of life, the functional adaptation of the molecular pathways in individual neurons is required to properly integrate external and internal factors. Disturbances in calcium homeostasis, resulting from a dysregulation of the ability to handle, store, and transfer information, contribute to the neuropathological processes in neurons, particularly after brain injury. The coordinated actions of all systems responsible for the maintenance and restoration of calcium homeostasis involve a complex interplay between functional proteins, including oncomodulin, caldendrin, calneuron, NCS-1, GAP-43, and CaM kinase II, as discussed here, which can act locally and globally at different molecular levels (Figure 3).

Triggering Ca^2+^ signaling through the activation of Ca^2+^-dependent kinases can control the expression of downstream specific genes, including the activity of transcription factors. Additionally, within the cell, Ca^2+^ signals can be directly or indirectly decoded by a set of Ca^2+^ sensor and/or -buffering proteins, modifying the spatiotemporal aspects of Ca^2+^ transients. The efficient integration of all pathways triggered by Ca^2+^ signaling, including immunological responses, can result in short- and long-lasting adaptive responses, enabling cells to survive if functional connections are established during regeneration processes. Over the past few years, significant progress has been made in elucidating the neuroprotective strategies implemented by numerous neuronal proteins. However, further studies are needed to fully understand all the processes that contribute to this, especially in terms of nerve repair and regeneration.
